# Morphological Deformities as Biomarkers in Fish from Contaminated Rivers in Taiwan

**DOI:** 10.3390/ijerph6082307

**Published:** 2009-08-21

**Authors:** Peter Lin Sun, William E. Hawkins, Robin M. Overstreet, Nancy J. Brown-Peterson

**Affiliations:** 1 Department of Aquaculture, National Pingtung University of Science and Technology, Nei Pu, Pingtung, Taiwan; 2 Gulf Coast Research Laboratory, The University of Southern Mississippi, P.O. Box 7000, Ocean Springs, MS 39566, USA; E-Mails: William.Hawkins@usm.edu (W.E.H.); Robin.Overstreet@usm.edu (R.M.O.); Nancy.Brown-Peterson@usm.edu (N.J.B.P.)

**Keywords:** tilapia, morphological deformity, biomarker, indicator, river pollution

## Abstract

Tilapia (*Oreochromis* spp.) were collected seasonally from four contaminated rivers in southwestern Taiwan for studies of morphological deformities that could be used as biomarkers of contamination. Morphological deformities found in tilapia were separated into 15 categories. Overall, the prevalence of deformities such as split fins, lower lip extension and gill deformities were significantly related to various water quality parameters, including low DO and high ammonium, lead and zinc concentrations. The persistence of tilapia in polluted waters and the development of a suite of morphological deformities suggest that tilapia can be used as sentinels of non-point source pollution in rivers.

## 1. Introduction

In contaminated waters, fish may exhibit whole animal, morphological, histopathological, cellular, organismic, or parasitic aspects of abnormalities, some of which can be used as biomarkers of contamination exposure [1–10]. The use of abnormalities in fish as biomarkers has become more prevalent in recent years [6,11–17]. Biomarkers in fish can provide a chronic indication the environmental condition than can more general and acute indices such as plankton analyses or water quality parameters. However, the cause-and-effect relationships between biomarkers and certain suspected pollutants cannot always be established [18].

Assessing morphological deformities is one of the most straightforward methods to study the effects of contamination on fish because of the ease of recognition and examination when compared with other types of biomarkers. Different types of morphological abnormalities have been reported in fish taken from contaminated waters, including fin erosion [1,19] skull deformation [20,21]; jaw deformities [21,22]; skeletal deformities such as lordosis, scoliosis, and kyphosis [6,22,23]; opercular deformity [6,24]; fin deformity [1,6,25]; lower lip protrusion [6,26]; gill deformity [9,12,27,28]; ocular disorders [29]; scale deformity and disorientation [6,25,30,31]; and neoplasia or hyperplasia [32].

The choice of a single fish species for use as a potential biomonitor to evaluate aquatic contamination must be considered from a practical standpoint. The target organism should be a nonmigratory species, abundant, and easy to collect [5]. Furthermore, it is advantageous for a sentinel species to be globally distributed and easy to maintain and breed in culture. Finally, the species should exhibit quantifiable reactions to contamination. Although not truly a single species, the tilapia used in this study actually constituted two species of the genus Oreochromis and a hybrid of them [6]. This complex can be considered resident in rivers of Taiwan and related complexes are common in many other tropical and subtropical waters worldwide. Tilapia are easy to catch, culture, and breed and can tolerate a wide range of water quality variations and harsh environmental conditions [6,33,34]. Furthermore, tilapia can exhibit various kinds of abnormalities in contaminated waters [6]. Thus, tilapia was chosen as the indicator species for this biomonitoring study. Based on the ubiquitous nature of tilapia the results of this study may apply equally to other areas of the world.

Since the late 1970s, Taiwan has become more industrialized and prosperous, but its capacity to treat waste and sewage has not kept pace with this advancement [35–41]. In particular, some smaller cities, factories, and farms often discharge wastes into the rivers or accumulate them along the river banks with little or no treatment. Rivers in southern Taiwan are affected by various contaminants, including domestic wastes, animal husbandry wastes, agricultural pesticides, dioxins, PCBs, and heavy metals [35,37–42]. The majority of the research in the southern portion of Taiwan has focused on the Era-Jiin River as the most visibly polluted river [38,43], but other rivers in southern Taiwan also receive significant agricultural and industrial effluents [35–37,42,44]. To evaluate if morphological abnormalities in tilapia can be used as biomarkers, and if the species complex can be used as a bioindicator, we collected tilapia from four major rivers in southern Taiwan, the Tongkong, Kao-Ping, Era-Jiin, and Lin Bien rivers, from autumn 1994 through spring 1996.

## 2. Materials and Methods

### 2.1. Sampling Protocol

**Figure 1.**
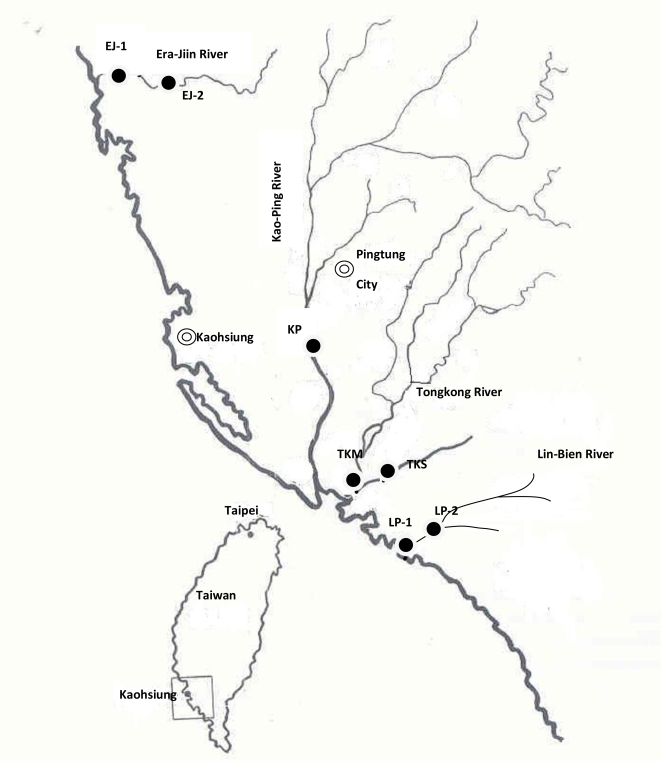
Location of Collection Stations on the Four Rivers Sampled in Southern Taiwan.

Tilapia (*Oreochromis* spp.) were collected from six stations in four rivers in southern Taiwan (Figure 1). The Kao-Ping River had one collection station (KP), a tidal freshwater station approximately 18 km upstream from the river mouth. There were two collection stations in the Tongkong River, a mesohaline location 1.5 km from the river mouth (TKM) and a tidal freshwater station located 5.3 km upstream from the river mouth at the Kon-She Dam (TKS). The Era-Jiin River also had two collection stations, a mesohaline station 6 km upstream of the river mouth (EJ1) and a tidal freshwater station 10.2 km upstream of the river mouth (EJ2). There was one tidal freshwater collection station in the Lin-Bien River (LB), 5 km upstream from the river mouth. Tilapia were collected seasonally during autumn (September–December) of 1994, spring (March–April) and autumn (September–October) of 1995, and spring (April–May) of 1996. Collecting times were associated with the beginning (spring) and end (autumn) of the rainy season. Adult tilapia were collected from shallow areas of the rivers near vegetation using gill nets (8- to 10- cm stretch) or by setting gill nets across the river. Gill nets were left in the water for no longer than 1 h. At least 30–40 adult fish per station per season were targeted for this study, but the actual number varied with the availability of fish. Juvenile tilapia (fry and fingerlings) were collected along the banks of the Kao Ping River during May 1996 and March–April 1997 by using a lift net baited with shrimp feed. Taxonomic identification of representative adult tilapia specimens from each station was provided by M.L.J. Stiassny, American Museum of Natural History, New York. Representative juveniles were identified in the laboratory of Dr. K.T. Shao, Institute of Zoology, Academic Sinica, Taipei, Taiwan by Dr. Chang Hao-Cheng.

### 2.2. Water Quality Parameters

Water quality parameters, such as dissolved oxygen (DO; mg/L), ammonia (mg/L), biological oxygen demand (BOD; mg/L), turbidity (NTU), suspended solids (mg/L) and the heavy metals chromium (Cr; mg/L), copper (Cu; mg/L), zinc (Zn; mg/L), and lead (Pb; mg/L) were obtained from representative sites close to each collection site from the Environmental Protection Department of Taiwan Province [45], and the data for mercury (Hg, mg/L) [46–48] and coliform bacteria (MPN/100 mL) were from various other sources. Monthly discharge values into the four rivers during 1994 and 1995 were obtained from the Water Resources Bureau [49] and the Department of Environmental Protection, National Pingtung University of Science & Technology [50]. Salinity, temperature and DO values were regularly measured during sample collections.

### 2.3. Morphological Examination

Adult fish were examined grossly for morphological abnormalities in the field. Gill opercula were opened to check for gill deformities. Those fish with notable morphological deformities were photographed with a Nikon FM-2 camera in the field and then preserved in 15% neutral buffered formalin. Fish suspected to have skeletal abnormalities were placed on ice and taken to the laboratory for radiographs using a Circlex 1.2 UGIOBN (Japan) X-ray machine.

Categorization of the morphological deformities in juvenile tilapia was based on the method of Koumoundouros *et al.* [51]. Fish less than 2.5 cm total length (TL) were examined using a stereoscopic microscope and those >2.5 cm TL with the aid of a magnifying lens. Gills of the small fish were examined by cutting the operculum before examination. The scales of juvenile tilapia were examined grossly or with the aid of a microscope.

### 2.4. Statistical Analysis

Contingency tables were constructed for each category of morphological deformity allowing for analysis of differences between upstream and downstream stations in the same river, differences between spring and autumn collections within a river for each year, differences in upstream or downstream stations among rivers, differences between adult and juvenile tilapia, and differences among juvenile specimens. The G-test of independence with the William correction (Gadj; [52]) was used to determine the association of abnormalities across time, stations, life stages, and species. In cases where more than two variables resulted in a significant difference, unplanned tests of the homogeneity of replicates were calculated (GH; [52]). Stepwise multiple regression analysis was used to determine the relationship between selected morphological deformities and various physical and chemical water quality parameters. The percentage of deformities was arcsine square root transformed prior to regression analysis [52]. Regressions were calculated using SYSAT version 9 [53]. For all tests, results were considered significant when p < 0.05.

## 3. Results

### 3.1. Description of the Collection Sites

In Taiwan the rainy season accounts for 78% of the total annual rainfall for the whole island and 90% of the annual rainfall in the southern portion of the island. The island is narrow and mountainous in the central portion, so most of the rivers flow in a steep descent from the center of island to the sea. River flow is high during the rainy season (May through September) but they typically flow at a lower rate during the dry season (October through April). The population is concentrated in the middle and lower sections of the rivers because the upper reaches are mostly mountainous areas. Most towns in the drainage areas of the four major rivers have populations not exceeding 50,000 or 60,000. The only large city is Pingtung City on the Kao-Ping River, with a population of 200,000.

The physical-chemical characteristics of the four rivers and their sampling stations are listed in Table 1. The Kao-Ping River is the largest river in southern Taiwan and the second largest river in Taiwan. The Era-Jiin River has a much smoother slope compared to the other three rivers, resulting in a slower water flow. The Lin Bien River has the steepest slope of the four rivers, resulting in rapid flow of water into the ocean after rain events. However, some water remains in the river bed even during the dry season. The Era-Jiin River has a muddier bottom than the Kao-Ping, Tongkong, and Lin Bien Rivers. Thus, suspended solid and the annual sediment discharge are considerably higher in the Era-Jiin than in the other three rivers, even though turbidity is often higher in the Lin Bien than the Era-Jinn River, particularly during heavy rains. Overall, the Tongkong River has the lowest sediment load in its waters, based on turbidity, suspended solids, and annual sediment discharge. All sampling stations were relatively shallow, with the deepest water (3.3 m) occurring at the TKM station.

During 1995, the rainy season started two months later than usual, resulting in lower annual rainfall. As a result, the mean discharge in all four rivers was approximately 50% lower than the mean discharge in a normal year. Consequently, there was less flushing through the river channels during the summer of 1995 [49].

The major pollution sources of the four rivers are summarized in Table 2. Animal wastes, mainly from swine farming, and to a lesser extent duck, chicken, and fish farming, were the main pollution sources along these rivers, with untreated animal wastes frequently seeping directly into the waters. Industrial pollution was also a significant source of contamination in southern Taiwan, as many factories discharged their sewage with limited treatment directly into the rivers. Discharge into the Kao-Ping River occurred mainly in the Pingtung City and surrounding areas due to the prevalence of factories in that area. The heavy metal refinery industry along the Era-Jiin River was a major source of pollution and PCBs and dioxin were also discharged directly into that river. Industrial pollution was not as prevalent in the Tongkong River, and there was little industry along the Lin Bien River with the exception of a few gravel mining operations. Domestic sewage was deposited into waste collection pits along the banks of all the rivers and some flowed into the rivers during the rainy season. Additionally, non-point source pollution in the form of waste and garbage was littered along the river banks and some entered the water during a heavy rainfall, resulting in decomposition and contamination of the river water. Finally, coastal seawater contamination could also impact the rivers, particularly in downstream portions. In general, there were fewer pollution sources along the Lin Bien River than the other three rivers, and this river was rated —unpolluted” by the Taiwan EPA. Thus, the Lin Bien was initially considered to be relatively unpolluted when compared with the other rivers sampled.

Water quality values for the four rivers are summarized in Table 3. Heavy metals listed in Table 3 were the only ones that were measured, although many other contaminants likely occurred in the rivers. The Era-Jiin River was the most seriously contaminated Taiwan river with regard to heavy metals, including aluminum. In the Lin Bien River, most of the measured heavy metals exceeded the EPA maximum recommended values, even though there was little industrial development along this river. The high value of metals is likely related to unlawful dumpling of wastes along bank [56]. Occasionally, higher values for metal concentrations than those reported here may occur in all rivers due to heavy rains or winds stirring up the bottom and re-suspending contaminated sediments. In all four rivers, discharge from swine farming was the major origin of the high ammonia, high coliform counts, high BOD, and low DO values. Although swine farming occurred along the upper and middle sections of the Lin Bien River, the ammonia concentration and coliform counts were lower than in the other rivers, and the DO values usually were much higher (Table 3). Organic pollutants were prevalent in all four rivers. Dioxin contamination was detected at all six stations, as well as PCB contamination, particularly in the Era-Jinn and Kao-Ping rivers [38]. These may be related to the presence of a heavy metal refinery or to acid washing work along these rivers [38,43,57].

### 3.2. Classification of Morphological Deformities and Their Rates of Occurrence

Morphological deformities of adult tilapia were separated into 15 categories that described abnormalities on all portions of the fish. These categories were: (1) split fins, (2) scale disorientation (including thickened and deformed scales), (3) hyperplasia of the surface of the mouth, (4) muscle atrophy, (5) opercular deformity, (6) gill deformity (including gill raker, gill arch, and gill filament deformities), (7) eye deformity (including the subcategories of exopthalmia, concave eye, small eye, blindness, lens deformity, and opaque cornea), (8) skeleton deformity (including vertebral and skull deformities), (9) outward protrusion of the lower lip, (10) tumors and other swellings, (11) jaw deformity (including one or two sides of the jaw having raised calcifications), (12) head or lower jaw bent to one side, (13) protruding mouth or nose part depression, (14) fin deformity (including elongated fin, part of the fin missing, and fin ray deformity), and (15) miscellaneous items (including body shape deformity and protrusion of the mandibular cartilage).

Table 4 shows the percentage of fish captured in autumn 1994 for each category of morphological deformity for each of the six stations. Autumn 1994 was the only collection period when tilapia were obtained from all six stations. Repeated attempts to collect in the Era-Jiin River during 1995 and spring 1996 yielded no tilapia. Collections were discontinued in the Lin Bien River after autumn 1994 based on water quality results and the high percentage of deformities found. This station had originally been identified as a control reference site.

During autumn 1994 fish in all rivers exhibited a wide variety of morphological deformities (Table 4). Deformities that occurred at all stations during autumn 1994 included split fins and gill deformities. Other common deformities were scale disorientation, opercular deformities, opaque cornea, and jaw deformities. Deformities that were least prevalent included hyperplasia on the surface of the mouth, muscle atrophy, exophthalmia, concave and small eyes, blindness, malformed lens, and distorted body shape. Even though tilapia collected from all rivers displayed at least six different categories of morphological deformities, the percentage of fish representing each deformity category was generally low with the exception of split fins.

**Figure 2.**
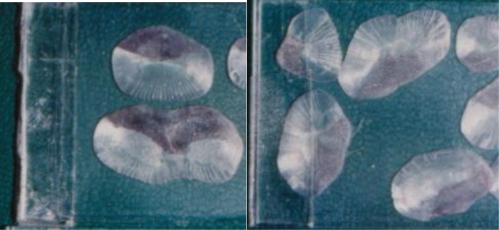
Abnormal Scales Collected from Tongkong River Mouth. Scales collected in October, 1994. These scales were not only deformed, but histologically the stratum corneum was thickened as well.

**Figure 3.**
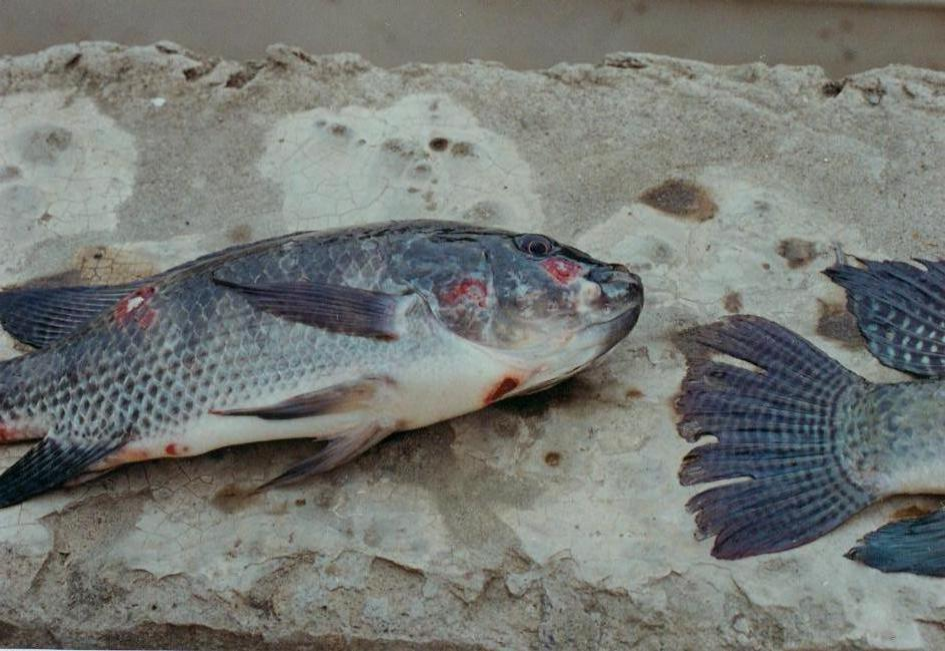
Tilapia with Body Lesions and Necrosis and Split and Lesioned Fins. Fish were collected from EJ-2 station of Era-Jiin River in October, 1994.

**Figure 4.**
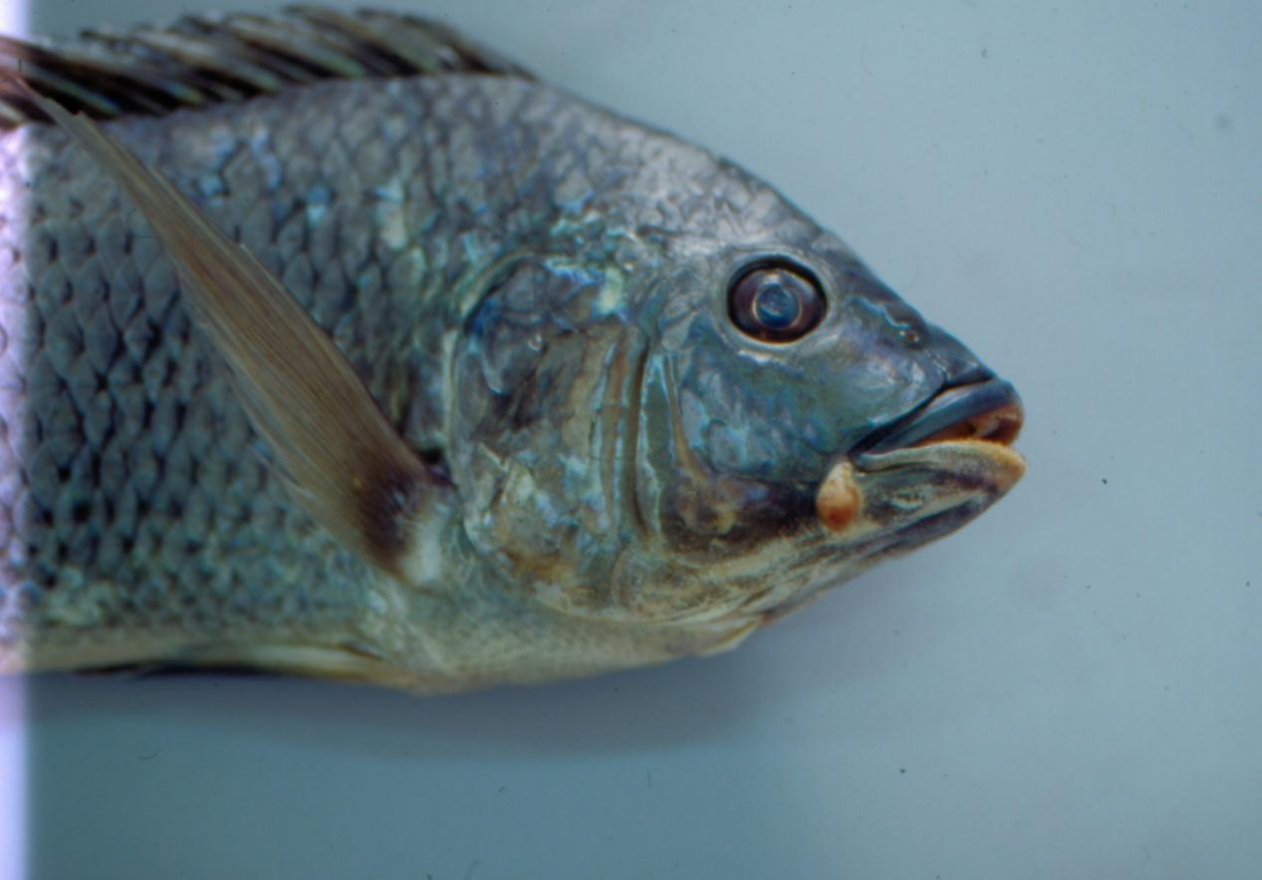
Tilapia with Mass on One Side of Mouth.

**Figure 5.**
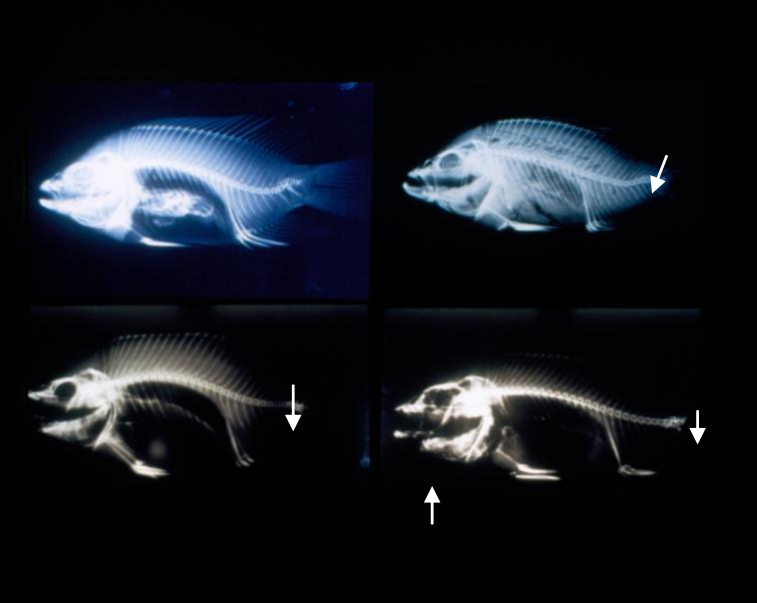
Different Types of Skeletal Deformities in Tilapia. Upper left: X-ray of tilapia collected from Lin-Bien River in October, 1994. Arrow shows vertebral segments close to tail are fused, calcified and deformed. Lower left: X-ray of tilapia collected from TKS station of Tongkong River in October, 1994. Note fusion of vertebrae near the tail (arrow). Upper right: X-ray of tilapia collected from the mouth of the Tongkong River in May, 1995. Note lordosis near the tail (arrow). There was also muscular atrophy in the tail area as well. Lower right: X-ray of tilapia collected from Kao-Ping River in October 1994. Arrow shows heavy calcification in the lower jaw, and fusion of vertebrae near the tail.

**Figure 6.**
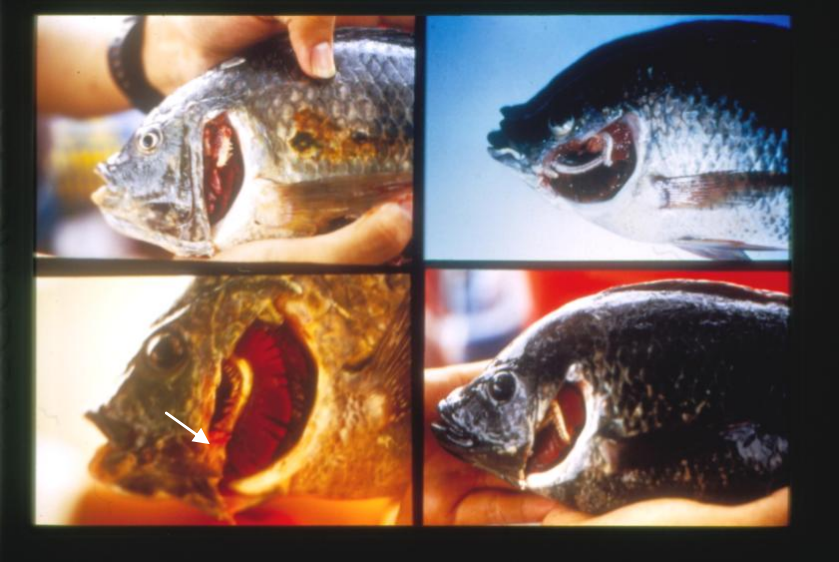
Gill Deformites. Upper Left: Gill arch deformities in tilapia collected from Tongkong River mouth. Lower Left: Gill rakers deformities (arrow) in tilapia collected from TKS station of Tongkong River. Upper Right: Gill arch deformities in tilapia collected from Tongkong River mouth. Lower Right: Gill arch, gill filament and gill rakers deformities in tilapia collected from TKS station of Tongkong River. All samples collected in September, 1995.

The 15 morphological categories described above can be reduced into six general abnormality groups: scale disorientation, gill deformity, eye deformity, skeletal deformity, soft tissue deformity, and split fins. The skeletal deformity group includes opercular, vertebrate, skull and jaw deformity, head or lower jaw bent to one side, protruding mouth or nose part depression, body shape deformity, mandibular cartilage protrusion, and fin deformity. The soft tissue deformity includes hyperplasia on the surface of the mouth, muscle atrophy or body shrinkage, lower lip protrusion, and tumor-like proliferation. Table 5 shows the percentages of fish captured from each of the six stations in the six general deformity classes. When deformity categories are combined, split fins and skeletal deformities were the most frequent types of morphological abnormality and eye deformities were the least common. Photos of some deformed tilapia are shown in Figures 2 to 6.

### 3.3. Seasonal Differences in Morphological Deformities

Seasonal collections were restricted to the Tongkong and Kao Ping rivers. Table 6 shows the percentages of each category of morphological deformity for each season at the three stations. Two-way contingency table analysis showed a significantly higher percentage of split fins at TKS in autumn 1994 than in spring 1995 (Gadj = 18.04, p < 0.001), although a comparison of the same seasons for 1995–1996 was not significantly different. However, when data from autumn of 1994 and 1995 were combined, there was a significantly greater percentage of split fins than in spring 1995 and 1996 combined (Gadj = 20.708, p < 0.001).

A similar pattern was seen downstream in the Tongkong River; the percentage of split fins was greater in autumn 1995 than in spring 1995 (Gadj = 11.144, p < 0.001) at TKM. The results from the Kao Ping River were the same as those from TKS. The percentage of tilapia with split fins was significantly higher in autumn 1994 than spring 1995 (Gadj = 9.18, p < 0.001) as well as in autumn 1994–1995 when compared with spring 1995–1996 (Gadj = 7.66, p < 0.01). Thus, tilapia from both river systems appear to have a significantly higher percentage of split fins in autumn than in spring. The autumn sampling period was at the end of the rainy season, which corresponded with increased river flow and sediment suspension. Tilapia with frayed fins, a condition not as severe as split fins, were also commonly observed throughout the course of this study in both the Kao-Ping and Tongkong Rivers (Table 7). Similar to findings with split fins, the percentage of fish with frayed fins was significantly greater during the rainy season than the dry season at all three stations (Table 7).

Several other non-grouped categories for morphological deformity also showed seasonal differences. Tilapia had a greater percentage of both gill and opercular deformities in autumn 1995 than in spring of that year at the TKM station (gill Gadj = 8.316, p < 0.005; opercular Gadj = 4.332, p < 0.05). There were numerous seasonal differences between autumn 1994 and spring 1995 at the KP station. Tilapia at KP also had a greater percentage of opercular deformities during autumn 1994 than spring 1995 (Gadj = 4.475, p < 0.05), similar to the situation at TKM. Tilapia at the KP station had a significantly higher percentage of opaque cornea in spring 1995 *vs*. autumn 1994 (Gadj = 8.163, p < 0.005). In contrast, tilapia had a higher percentage of opaque cornea in autumn 1995 than spring 1996 at KP (Gadj = 15.773, p < 0.001). There was no seasonal difference in cornea opacity when data from both spring and autumn samples were combined. However, the data indicate severe problems with opaque cornea at the KP station during all of 1995. The mandibular cartilage protrusion deformity was seen in tilapia from KP only and was observed during spring 1995 only (Table 6). There was no other statistically significant difference in seasonal occurrence of morphological deformities at TKS, TKM, or KP.

### 3.4. Comparison of Morphological Deformities among Stations

Collections were taken from both river mouth and upstream stations in the Tongkong and Era-Jiin Rivers. Additionally, supplemental collections were taken from a downstream and an upstream station in the Ssu-Chung River during autumn 1994. Overall, there was no difference in the percentage of morphological deformities in any category or in any season for any of the three rivers when compared with either downstream or upstream stations. The only exception was that tilapia at TKM (downstream) had a significantly higher percentage of skeletal deformity (Gadj = 4.63, p < 0.05) than those at TKS when data from all seasons were combined.

A comparison of morphological deformities during autumn 1994 in the tidal freshwater stations (KP, TKS, EJ2 and LB) showed a significantly higher percentage of split fins in the Era-Jiin River than in all other rivers (Gadj = 64.25, p < 0.001; Table 4); there was no difference in percentage of split fins among KP, TKS and LB. A comparison of morphological deformities during autumn 1994 in the mesohaline stations (TKM, EJ1) also showed significantly higher parentage of split fins in EJ1 than TKM (Gadj = 5.79, p < 0.005). When all stations and all seasons were combined for analysis, the percentage of split fins was significantly higher in EJ1, EJ2 and LB than at the other three stations (Gadj = 63.44, p < 0.001; Table 5). Furthermore, TKM showed a significantly lower percentage of split fins than the other five stations (Gadj = 27.86, p < 0.001). While a comparison among all six stations with data from all seasons combined showed no significant difference in the percentage of split fins between TKS and KP, when just these tidal freshwater stations were compared, KP had a significantly higher percentage of split fins than TKS (Gadj = 7.89, p < 0.005). There was also a significantly higher percentage of split fins at KP than at TKS during spring 1995 (Gadj = 4.91, p < 0.05; Table 6), although there was no other season that showed a significant difference.

The percentage of tilapia showing scale disorientation was significantly lower in the Lin Bien River during autumn 1994 when compared to the other three tidal freshwater stations (Gadj = 7.10, p < 0.05); there was no difference in percentage of scale disorientation among EJ2, KP, and TKS. When data from all seasons were combined and all six sites were compared, there was a significantly higher percentage of scale disorientation in EJ2 and TKM than in LB (Gadj = 18.82, p < 0.005). The percentage of scale disorientation was not significantly different when TKS, KP, and EJ1 were compared with LB or with EJ2 and TKM.

The prevalence of opaque cornea was significantly higher in KP than in all other rivers (Gadj = 32.9, p < 0.001) when seasonal data were combined. During spring 1995, the percentage of fish with opaque cornea was significantly higher in KP than TKS (Gadj = 4.3, p < 0.05). However, there was no significant difference between the two stations in autumn 1995 when a high percentage of fish in both areas exhibited opaque cornea (Table 6).

Tilapia with skeletal deformities such as those of the vertebrae, operculum, fin, head, and jaw showed two distinct groupings by station when data from all seasons were combined. Tilapia from EJ1, TKM, and KP had a significantly higher percentage of skeletal deformities than those from TKS, LB, and EJ2 (Gadj = 22.04, p < 0.001). However, there was no significant difference among stations in either the high group (EJ1, TKM, KP) or the low group (TKS, LB, EJ2).

Tilapia from the Lin Bien River had a significantly lower percentage of soft tissue deformities than those from the four stations in the Era-Jiin and Tongkong rivers (Gadj = 22.36, p < 0.001) when data from all seasons were combined. However, there was no significant difference in soft tissue deformities between LB and KP or between KP and the other four stations. The KP station also had the lowest percentage of tilapia with gill deformity when data from all seasons were combined. Station EJ1 had a significantly higher percentage of gill deformity than KP (Gadj = 22.75, p < 0.001). However, there was no significant difference among EJ1, EJ2, TKM, or LB for gill deformity. Similarly, the percentage of gill deformity was not significantly different among KP, TKS, LB, TKM, and EJ2.

### 3.5. Relationship between Morphological Deformities and Water Quality

The relationships among the six most common morphological deformities and water quality parameters were examined using multiple regression techniques. Water quality parameters available for these analyses included DO, ammonia concentration, biological oxygen demand (BOD), total suspended solids, and concentrations of the heavy metals Cu, Cr, Pb, Hg and Zn.

The percentage of tilapia exhibiting split fins was negatively correlated with DO and ammonia concentration (r2 = 0.855, p < 0.001) and positively correlated with Zn concentration (r2 = 0.407, p = 0.014). However, when these three parameters were combined into the same regression equation, only DO and ammonia showed a significant relationship with split fins. The percentage of tilapia with scale disorientation was negatively correlated with suspended solids (r2 = 0.391, p = 0.017); none of the other parameters had any relationship to scale disorientation. There was a negative correlation between the percentage of tilapia with gill deformities and the combination of DO and ammonia (r2 = 0.714, p = 0.002). Gill deformity also showed a positive relationship to Pb concentration and a negative relationship to Hg concentration (r2 = 0.552, p = 0.012); when DO and ammonia were added as additional variables to this equation, they did not increase the correlation. The relationship between lower lip extension and DO was negative (r2 = 0.642, p = 0.001), and there was a positive relationship with Pb concentration and a negative relationship with Zn concentration (r2 = 0.506, p = 0.021). However, the addition of the heavy metal variables to the DO variable did not increase the correlation with lower lip extension. Finally, the percentage of fin deformities was negatively related to the concentration of Cr (r2 = 0.365, p = 0.022); no other variable significantly affected fin deformities. There was no relationship between the percentage of opercular deformities and any of the water quality variables.

Overall, the results of these analysis suggest that a suite of morphological deformities in tilapia, including fin splits, lower lip extension and gill deformities is related to poor water quality, as indicated by low DO, high ammonia concentration, and high concentrations of Pb and Zn. Interestingly, fin deformities and scale deformities appear to increase with decreasing concentrations of Cr and suspended solids, respectively, although neither of these relationships is highly predictive (r2 < 0.4).

### 3.6. Morphological Deformities of Juvenile Fish in Kao-Ping River

Juvenile tilapia collected from the Kao-Ping River during the dry seasons of 1996 and 1997 were compared with adults collected during the spring of 1995 and 1996 and autumn of 1995. Table 8 shows the percentages of juvenile and adult tilapia with seven different morphological deformities. The most common morphological deformity categories for the juveniles were frayed or deformed fins, while the most common categories for the adults were frayed or elongated fins. Although a greater percentage of juvenile fish had head, vertebral or fin deformities than adults, there was no significant difference in these deformity categories between groups. However, a significantly greater percentage of adult tilapia had frayed fins and elongated fins, compared with juveniles (frayed fins Gadj = 4.04, p < 0.05; elongated fins Gadj = 8.99, p < 0.05) (Table 8). There was no other significant difference in morphological deformities between juveniles and adults in the Kao-Ping River.

Morphological deformities of juvenile tilapia were compared with those of juveniles of three other species (topmount gudgeon, *Pseudorasbora parva*: common carp, *Cyprinus carpio*; spotbanded gobi, *Rhinogobius maculafasciatus*) collected concurrently from the Kao-Ping River during March and April 1997 (Table 9).

Tilapia had more categories of morphological deformities than did the other three species. However, the highest percentages of deformed juvenile tilapia were found in just three categories (split fins, skeletal deformity, and fin deformity) which corresponded with the three main categories of deformities for the other species collected (Table 9). No juvenile of any species was found with scale disorientation, hyperplasia on the surface of the mouth, gill deformities, outward extension of the lower lip, tumorous protrusions or jaw deformities. These morphological abnormalities might develop later in life, as evidenced by the high percentage of some of these categories in adult tilapia (Table 6). Additionally, opercular deformity, eye deformity, head or lower jaw bent to one side, protruding mouth, and body shape deformities occurred in juvenile tilapia only but were rare. There was no significant difference in the split fin or skeletal deformity categories among the four juvenile species, although there was a significant difference in fin deformity (Table 9). However, there was no significant difference in fin deformity among *C. carpio*, *R. maculafasciatus* and Tilapia. (GH = 0.435, p > 0.05).

## 4. Discussion

In southern Taiwan, large factories, such as those in industrial parks, are controlled by national regulations, but many family-owned factories as well as swine farms unlawfully discharge their sewage directly into rivers. Also, wastes are deposited along rivers side or directly dumped into those rivers. These situations have made many rivers of southern Taiwan such as the Tongkong, Kao-Ping and Era-Jiin Rivers become highly polluted. Overall, this study strongly suggests that the types and degrees of non-point source contamination together with physical and chemical condition of each sampling station likely resulted a variety of morphological deformities in resident tilapia. The morphological deformities represented by 15 categories with each category further subdivided. For example, scale disorientation included scale deformity and thickened scales, fin deformity included abnormal fin ray, elongated or unpaired fin, incomplete fins and extra fin ray grow at the fin base. Furthermore, the degree of deformity varied within sub-category resulting in low prevalence in most sub-categories. Compared with the variety of categories of deformity caused by non-point source pollution as seen in the present study, the categories of deformity caused by pollution say from a single large factory [21,23], or by a particular pollutant [9,25,51] are usually much less, although the incidences of deformity are typically higher.

The Era-Jiin River, especially in its lower section, was considered the most heavily polluted river in Taiwan mainly by heavy metals as a result of acid wash procedures, refinery operations, and organic pollution [45]. The lower sections of both the Kao-Ping and Tongkong Rivers are rated as moderately polluted but the Kao-Ping River was more heavily polluted by heavy metals but less by organics than was the Tongkong River. The Lin Bien River was rated as unpolluted river by the Taiwan EPA [45] with much less organic pollution than the other three rivers, but was affected by heavy metals possibly due to the waste disposal sites located along river and industrial wastes being dumped into the river. Furthermore, the Lin Bien river bed often is devoid of water in the dry season because its slope is steep (1:15), causing pollutants to accumulate in river bed during dry season. But after heavy rain falls, those pollutants are mobilized resulting in high concentration of heavy metals in the river. All six collecting stations were considered to be in the lower section of each of the four rivers studied. In addition to heavy metal and organic pollution, various other pollutants appeared at different times and sections of the four rivers.

Based on the statistical analyses of the percentage of deformities of all stations, the Era-Jiin River appears to be the most seriously polluted of the four rivers and Lin Bien River the least. When data on deformities from fish collected in all seasons were pooled and when those from the two stations from the Era-Jiin River were pooled, Era-Jiin River appears to be the area most affected by contamination and the Lin Bien River appears the least affected. Additionally, EJ1 had a significantly higher percentage of gill deformity than KP. The significantly higher morphological deformities of fish from stations of Era-Jiin River, especially the EJ1 station is probably related to heavy metal contamination, because there were many small family run heavy metal refinery plants along the river. On the other hand, statistical analyses showed the LB station had the lowest percentage of morphological deformity. Scale disorientation in the LB station was significantly lower than EJ2 and TKM and soft tissue deformities were significantly lower than those from the four stations in the Era-Jiin and Tongkong Rivers. Tilapia at TKM had a significantly higher percentage of skeletal deformity than did those at TKS when data from all seasons were combined. Prevalence of morphological deformities at stations of Tongkong and Kao-Ping River were between those of the Era Jiin and Tongkong Rivers. Thus, it appears that morphological deformities reflect the water quality of these rivers.

Scales collected from TKM tilapia in 1994 were not only deformed but histologically the stratum corneum of scales was thickened as well. Changes in salinity should not affect scales but might exacerbate the effects of heavy metal and petroleum pollution and cause increased scale thickness and deformities. Near the mouth of the Tongkong River is a large fishing harbor that might contribute to organic (petroleum) pollution of TKM, station and heavy metal pollution has also increased along the coast from Kaohsiung city [57]. Both of those factors might have contributed to the thickened and abnormal scales of tilapia at the TKM station.

Morphological deformities such as those of fins, gills and opercula were significantly higher in fish collected in the rainy season than in the dry season. This might reflect river and rainfall characteristics since about 90% of the rainfall in southern Taiwan occurs during the rainy season. This increased river flow stirs up organic material and heavy metals from river bed and usually results in significant fish mortality in river mouth each year after the first heavy rainfall of the beginning of rainy season. It is likely that this situation contributes to the formation of deformities.

The rainfall for 1995 was much less than usual but 1994 was above normal resulting in a major difference of the annual main discharge between those two years and might have resulted in the increase in deformities, particularly split fin seen in the spring of 1995. The prevalence of those lesions was significantly less than for autumn 1994 for TKS. Furthermore, there was a higher prevalence of opaque cornea in 1995 than in 1994 or 1996 for both KP and TKS stations. Table 10 shows the annual main discharge (CMS) for 1994 and 1995 for those stations close to our collection sites [49].

Comparison of morphological deformities between juvenile and adult tilapia in the Kao-Ping River showed that only frayed fins and fin elongation were significant higher for adult tilapia but head, vertebral and fin deformities in juvenile tilapia were much higher than in the adult ones. Since these deformities might affect survival the rate would be much less in the adults than in juveniles. For scale disorientation, soft tissue deformity, and fin elongation, these occurred more frequently in adults than in juveniles as they tend to occur later in life [26]. For the Kao-Ping River in spring, 1997, juvenile tilapia had more categories of morphological deformities than did juvenile specimens of the other three species collected in that river. This adds further support to the utility of tilapia as a bioindicator species for contaminated waterways.

Tilapia collected from all stations showed external lesions on the body and fins and bleeding on lateral line or nose. We found that 35% of the tilapia collected from Tong-Kong River and 46% from Kao-Ping River had body or fin lesions or external areas of necrosis [58]. Histologically, macrophage aggregates and granulomas were found in liver and spleen or other tissues collected from all locations [6]. These non-specific biomarkers are typically found in areas with organic or heavy metal contamination, low DO and with endocrine disrupting compounds [1,59]. Additionally, abdominal edema, fatty liver, liver necrosis, pale or discolored liver, liver atrophy, external gill parasites and abnormal blood smears were commonly found as well. These findings were supplementary evidence that the fish existed in compromised environments.

Both Kao-Ping and Tong-Kong Rivers have river weirs across the river that were built before this study began and that hinder tilapia movement downstream. The weir on the Kao-Ping River was located a few kilometers upstream of KP station and the one on Tong-Kong River was few hundred meters above the KS station. Tilapia, especially *Oreochromis niloticus*, usually stay between the collecting stations and river weir salinities are low. As for the Lin Bien River, the water level was usually low especially during dry season. Tilapia typically move around only after river flooding caused by heavy summer rainfall or after typhoons. So in the present study the morphological deformities of the collected tilapia should have reflected the pollution condition of the collecting site. The contaminated rivers sampled in this study were much less polluted before 1970, the beginning of the economic boom of Taiwan. In the early 1970s, heavy metal acid wash and refinery industries were initiated near the Era-Jiin River. The Kao-Ping River drainage basin was also industrialized in this period with the initiation of heavy metal industry. According to Hung *et al.* [41], heavy metal pollution increased substantially around 1970 in the Kao-Ping canyon, suggesting that the increase in metal pollution began before the economic boom of Taiwan. In the present study, tilapia generally lacked parasites required intermediate hosts suggesting that the food web was impaired by the environmental pollution in these rivers. This was most obvious for Era-Jiin River and there were no tilapia caught in this river after the 1994 collections [58]. Another study also pointed out that there were no tilapia in this river and suggested that all the sentinel fish species in this river were victims of heavy pollution [40].

Recently river pollution in Taiwan has improved somewhat, mainly because of regulations placed on the swine and heavy metal refinery industries. Nevertheless, Taiwan still has a long way to go before river water quality is acceptable [44].

Our findings clearly demonstrate that tilapia from these rivers that have different types and concentrations of pollutants such as heavy metals of Hg, Zn, Pb, Cu, Cr, serious organic pollution as evidenced by high ammonia levels, BOD, coliform counts and low DO along with high suspended solids with high flowing speed during heavy rainfall, developed a suite of morphological deformities in response to those pollutants. Meanwhile, this sentinel species apparently acquires more types of morphological deformities than many other fish species documented to date.

## 5. Conclusions

This study established that morphological biomarkers in tilapia (*Oreochromis* spp.) can be used as a good tool for studies involving non-point sources of pollution. Tilapia not only tolerant severe contaminated waters but show a variety of specific morphological deformities and lesions that appear to reflect the level of river pollution. Since several years have elapsed since this study was conducted, we recommend that a reanalysis of the health status of tilapia in southern Taiwan rivers be conducted to determine whether regulation and remediation efforts have been effective.

## Figures and Tables

**Table 1.** Physical-chemical Characteristics of the Four Major Rivers in Southern Taiwan and Their Sampling Stations.

**Table 1-1.** Physical Characteristics of the Four Rivers.						
**Characteristic**	**Kao-Ping River**	**Tong Kong River**		**Era-Jiin River**		**Lin Bien River**
River length (km)	170.9	46.90		65.18		42.19
Drainage area (km^2^)	3,256.8	472.20		350.4		344
Annual sediment discharge (MT/km^2^)	10,934	1,292		37,424		5,349
Mean annual runoff (10^6^ M^3^)	8,455.35	1,117.62		498.88		863.84
Slope	1:43	1:41		1:142		1:15
Average annual Rainfall (mm)	3,046.1	2,499.8		1,909.9		3,330.7
**Table 1-2.** Physical Characteristics and Salinity of Collection Stations.						
**Characteristic**	**KP**	**Tong Kong River**		**Era-Jiin River**		**LB**
		**TKS**	**TKM**	**EJ1**	**EJ2**	
River width (m)	300	150–200	600–700	200	150	300–350
Bottom type	Sand & stone	Sand & mud	Sand	Mud & sand	Mud	Sand & stone
Average depth (m)	1.5	2.5	2.0–3.3	2.0	1.5	0.5–1.5
Salinity	Tidal freshwater	Tidal freshwater	Mesohaline	Mesohaline	Tidal freshwater	Tidal freshwater
Discharge (m^3^/s)	7.2–1,000^a^	2.7–48.1^b^	9.0–66.7^b^			17.95

Source: [49]

Source: Measured *in situ* for this study;

^a^ [49];

^b^ [50].

**Table 2.** Main Pollution Sources of Four Contaminated Rivers in Southern Taiwan. The Percent Contribution to the Overall Pollution is Indicated for Each Source.

**Source**	**Tongkong River**^1^	**Kao-Ping River**^2^	**Era-Jiin River**^3^	**Lin Bien River**^4^
Animal Husbandry	Swine, chicken, duck, and fish farming—72%	Swine, chicken, duck, and fish farming—58%	Swine, duck, chicken, and fish farming—51%	Swine, duck, and chicken farming—70% Fish farm waste--22%
Industry	Industrial district (Food processing, fabric, metal industry, etc.)—11%	Leather industry heavy metal reclamation, food processing—31%	Heavy metal refinery, electrical processing, acid washing—38%	Few gravel factories—2%
Domestic	Sewage and garbage—12%	Sewage and garbage—8%	Sewage and garbage 10%	Sewage and garbage—6%
Seepage, Non-point source	5%	3.3%	<1%	<1%

Sources:^1^ [35];

^2^ [36];

^3^ [54];

^4^ [55].

**Table 3.** Range of Selected Water Quality Parameters of the Collection Stations from 1993–1996.

		**Era-jiin River**		**Kao-ping River**	**Tong-Kong River**		**Lin-Bien River**
		**EJ-1**	**EJ-2**	**KP**	**TKS**	**TKM**	**LB**
Suspended Solids (mg/L)		8.0–65*	8.5–120*	13–312*	5.5–83*	6.0–121*	18–5,700*
NH_4_^+^-N (mg/L)		0.64–34*	0.77–44*	0.16–6.1*	0.85–16*	0.11–14*	0.04–0.74*
DO (mg/L)		0–11*	0–10.7*	0–8.7*	0–5.5*	0–14*	0.69–9.2*
BOD (mg/L)		2.6–47*	3.3–68*	0.4–12*	3.0–23*	1.7–33*	0.1–6.9*
Coliform (MPN/100ML)		240,000*^a^		5,000–93,000*	30,000–1,600,000*	24,000–500,000*	240–240,000*
Heavy metals	Cd (mg/L)	<0.005–<0.01	<0.005–<0.05	<0.005–<0.01	<0.001–<0.01	<0.005–<0.01	<0.005–<0.01
	Cr (mg/L)	<0.001–0.19*	<0.001–0.29*	<0.01–0.11*	<0.01	<0.01	<0.01–0.08*
	Cu (mg/L)	0.04–0.19*	0.01–0.22*	<0.01–0.02	<0.01	<0.01–0.02	<0.01–0.11*
	Pb (mg/L)	0.001–0.04	0.01–0.111*	<0.01	<0.01	<0.01	<0.01–0.13*
	Zn (mg/L)	0.07–0.81*	0.6–1.0*	0.02–0.10	0.02–0.06	<0.01–0.08	0.02–2.2*
	Hg (μg/L)		15.94*^b^	<0.011–3*^c^	<0.001–4.0*^c^		<0.001–0.9^d^

* Indicated value exceeds maximum allowed value. Source: [45] (except the value with a superscript).

Sources of Hg values: ^b^[46];

^c^ [47];

^d^ [48].

**Table 4.** Morphological Deformities of Tilapia Collected from Six Stations in Four Rivers in Southern Taiwan in Autumn, 1994. All values expressed as percentages of total fish caught at each station. TKS–Tongkong River upstream station; TKM–Tongkong River mouth station; KP–Kao-Ping River station; EJ1–Era Jiin River downstream station; EJ2–Era-Jiin River upstream station; LB–Lin Bien River.

**Deformity**		**TKS**	**TKM**	**KP**	**EJ1**	**EJ2**	**LB**

Split fins		46.0	37.5	52.0	87.5	62.5	50.0
Scale disorientation		13.0	12.5	18.0	6.2	25.0	0
Mouth hyperplasia		2.0	0	2.0	0	0	0
Muscle atrophy		2.0	0	0	0	0	0
Opercular deformity		8.0	12.5	14.0	12.5	0	3.0
Gill deformity		2.0	25.0	2.0	31.2	16.7	3.0

Eye deformity	Exopthalmia	3.8	0	0	0	0	0
	Concave eye	0	0	0	0	0	0
	Small eye	0	0	0	0	0	0
	Blindness	1.9	0	0	0	0	0
	Lens deformity	0	0	0	0	0	0
	Opaque cornea	5.8	12.5	4.5	6.2	0	3.0

Skeleton deformity		6.0	0	0	0	0	3.0
Lower lip extension		2.0	12.5	0	31.3	16.7	0
Tumor or bump		2.0	0	0	0	4.2	0
Jaw deformity		13.0	0	7	37.5	16.7	6
Head or jaw bent to side		0	0	7.0	0	0	3.0
Mouth protrusion		2.0	0	0	0	0	0
Fin deformity		10.0	0	2.0	0	0	6.0
Miscellaneous		2.0*	0	0	0	0	0
Total number of fish		52	8	44	16	24	32

^*^ Body shape deformed.

**Table 5.** Morphological Deformities of Tilapia Collected from Six Stations in Four Rivers in Southern Taiwan, 1994–1996. All values are expressed as percentages of total fish caught at each station. Locations as indicated in Table 4.

**Deformity**		**TKS**	**TKM**	**KP**	**EJ1**	**EJ2**	**LB**

Scale disorientation		12.5	20.4	9.6	6.2	25.0	0
Gill deformity		2.6	7.4	2.4	31.2	16.7	3.0

Eye deformity	Exopthalmia	2.0	3.0	3.0	0	0	0
	Concave eye	0	1.0	1.0	0	0	0
	Small eye	0.5	0	0	0	0	0
	Blindness	0.5	3.0	0	0	0	0
	Lens deformity	0	1.0	0	0	0	0
	Opaque cornea	10.0	10.0	26.0	6.2	0	3.0

Skeletal deformity		28.5	46.3	37.1	50.0	16.7	21.8
Soft tissue deformity		12.9	13.0	3.0	31.3	20.8	0
Split fins		27.1	13.9	42.5	87.5	62.5	50.0
Total number of fish		192	108	167	16	24	32

**Table 6.** Morphological Deformities of Tilapia Collected Seasonally from the Tongkong and Kao-Ping Rivers in Southern Taiwan from 1994 through 1996. All Values Expressed as Percentages. A–autumn; S–spring.

**Deformity**		**Tongkong River (TKS)**				**Tongkong River (TKM)**			**Kao-Ping River (KP)**			

		**A/94**	**S/95**	**A/95**	**S/96**	**A/94**	**S/95**	**A/95**	**A/94**	**S/95**	**A/95**	**S/96**

Split fins		46.0	4.0	53.0	35.3	37.5	1.0	37.0	52.0	20.0	62.0	40.0
Scale disorientation		13.0	13.0	9.0	11.8	12.5	16.0	33.0	18.0	8.0	2.0	10.0
Mouth hyperplasia		2.0	0	0	0	0	0	0	2.0	0	0	0
Muscle atrophy		2.0	1.0	0	0	0	1.0	7.0	0	0	0	3.3
Opercular deformity		8.0	2.0	9.0	5.9	12.5	0	13.0	14.0	0	2.0	6.6
Gill deformity		2.0	1.0	6.0	5.9	25.0	0	23.0	2.0	4.0	0	6.6

Eye	Exopthalmia	3.8	0	3.1	0	0	1.4	3.3	0	0	11.9	0
	Concave eye	0	0	0	0	0	0	0	0	0	2.4	0
	Small eye	0	1.1	0	0	0	0	0	0	0	0	0
	Blindness	1.9	0	0	0	0	2.9	3.3	0	0	0	0
	Lens deformity	0	0	0	0	0	1.4	0	0	0	0	0
	Opaque cornea	5.8	4.4	31.3	17.6	12.5	8.6	20.0	4.5	37.3	54.8	0

Skeletal deformity		6.0	0	0	5.9	0	3.0	7.0	0	2.0	5.0	0
Lower lip extension		2.0	14.0	22.0	0	12.5	10.0	10.0	0	0	7.0	0
Tumor or bump		2.0	0	0	0	0	0	0	0	0	0	0
Jaw deformity		13.0	6.0	3.0	0	0	7.0	7.0	7.0	12.0	12.0	13.3
Head or jaw bent		0	4.0	0	0	0	4.0	23.0	7.0	4.0	0	3.3
Mouth protrusion		2.0	1.0	0	5.9	0	1.0	7.1	0	0	0	0
Fin deformity		10.0	11.0	9.0	5.9	0	14.0	33.0	2.0	6.0	29.0	30.0
Miscellaneous		2.0^1^	1.0^1^	0	0	0	0	3.0^1^	0	2.0^1^ 16.0^2^	0	3.3^1^
Total number of fish		52	91	32	17	8	70	30	44	51	42	30

^1^ Body shape deformed;

^2^ Outward protrusion of mandibular cartilage.

**Table 7.** Tilapia with Frayed Fins Collected from the Tongkong and Kao-Ping Rivers in Southern Taiwan. G_adj_–William’s Correction of G Test of Independence.

**Station and Season**	**% Frayed Fins**	**N**	**G_adj_**	***p***

TKS, Rainy TKS, Dry	48.8 9.1	84 108	19.41	<0.001
TKM, Rainy TKM, Dry	36.8 1.4	38 70	12.28	<0.001
KP, Rainy KR, Dry	57.0 27.2	86 81	7.66	<0.01

**Table 8.** Morphological Deformities of Adult and Juvenile Tilapia in Kao-Ping River. All Values Expressed as Percentage. G_adj_–William’s Correction of G Test of Independence.

**Deformity**	**Juvenile**	**Adult**	**G_adj_**	***p***

N	83	123	–	–
Scale Disorientation	0	7	3.97	>0.05
Opercular Deformity	2	2	0.055	>0.005
Head or Vertebrae Deformity	10	3	1.72	>0.05
Frayed Fins	21	39	4.04	<0.05
Fin Deformity	18	6	3.82	>0.05
Fin Elongation	0	14	8.99	<0.05
Soft Tissue Deformity	0	4	2.36	>0.05

**Table 9.** Comparison of the Morphological Deformities between Juvenile Tilapia & Juveniles of Other Species Collected in Kao-Ping River in Spring, 1997. All Values Expressed as Percentages.

Spp. Item	*Pseudorasbora parva*	*Cyprinus carpio*	*Rhinogobius maculafasciatus*	*Tilapia spp.*

	13 March, 1 April 1997	1 April 1997	13 March 1997	13 March, 1 April 1997
Split fins	6.1	12.5	10	14.5
Scale disorientation	0	0	0	0
Mouth hyperplasia	0	0	0	0
Muscle atrophy	0	0	10	0
Opercular deformity	0	0	0	1.61
Gill deformity	0	0	0	0
Eye deformity	0	0	0	1.61
Skeleton deformity	8.2*	0	10	12.9
Lower lip extension	0	0	0	0
Tumor or bump	0	0	0	0
Jaw deformity	0	0	0	0
Head or lower jaw bent to side	0	0	0	1.61
Mouth protrusion	0	0	0	1.61
Fin deformity	0	19	30	16.1
Miscellaneous	0	0	0	1.61
Total number collected	49	16	10	62

* These four fish were all vertebrate deformed.

**Table 10.** Comparison of Historical Annual Main Discharge among Selected Stations for 1994 and 1995.

**River**	**Station**	**Historically**	**1994**	**1995**

Era-Jiin	Ah Lian (2)	9.22 CMS	13.79 CMS	5.34
Kao-Ping	Lee Ling	184.51 CMS	270.71 CMS	109.52
Tong-Kong	Chao Chou	16.41 CMS	18.80 CMS	8.69

Source: [49]
